# Immunomodulatory Effects of Anesthetics during Thoracic Surgery

**DOI:** 10.1155/2011/317410

**Published:** 2011-10-27

**Authors:** Khaled Mahmoud, Amany Ammar

**Affiliations:** Department of Anesthesiology, Faculty of Medicine, Minoufiya University, 3 Yaseen Abdelghaffar Street, Shebin Elkoam, Minoufiya 32511, Egypt

## Abstract

*Background*. One-lung ventilation (OLV) during thoracic surgery may induce alveolar cell damage and release of proinflammatory mediators. The current trial was planned to evaluate effect of propofol versus isoflurane anesthesia on alveolar and systemic immune modulation during thoracic surgery. *Methods*. Fifty adult patients undergoing open thoracic surgery were randomly assigned to receive propofol *(n* = 25) or isoflurane *(n* = 25) anesthesia. The primary outcome measures included alveolar and plasma concentrations of interleukin-8(IL-8) and tumour necrosis factor-*α* (TNF-*α*), whereas secondary outcome measures were alveolar and plasma concentrations of malondialdehyde (MDA), superoxide dismutase (SOD), and changes in alveolar albumin concentrations and cell numbers. *Results*. Alveolar and plasma concentrations of IL-8 and TNF-*α* were significantly lower in the isoflurane group, whereas alveolar and plasma concentrations of MDA were significantly lower in the propofol group. Alveolar and plasma SOD levels increased significantly in the propofol group whereas they showed no significant change in the isoflurane group. Furthermore, the isoflurane group patients developed significantly lower alveolar albumin concentrations and cell numbers. *Conclusion*. Isoflurane decreased the inflammatory response associated with OLV during thoracic surgery and may be preferable over propofol in patients with expected high levels of proinflammatory cytokines like cancer patients.

## 1. Introduction

One-lung ventilation (OLV) during thoracic surgery may trigger alveolar cell damage and release of proinflammatory mediators that might lead to lung injury and infection in the postoperative period [1]. A series of clinical and experimental studies on mechanical ventilation reported alteration of alveolar and systemic immunity during surgery and anesthesia [2–4]. Different factors have been implicated that include preoperative smoking or drugs, degree of surgical trauma, preexisting lung or systemic diseases, in addition to type and duration of anesthesia [5, 6].

Propofol has been suggested to suppress pathologic changes associated with acute lung injury during endotoxemia in rabbits [7]. Other experimental studies have reported that volatile anesthetics may alter cytotoxic or phagocytic activity of alveolar macrophages. [8] Both sevoflurane and desflurane have shown ant-inflammatory effect during thoracic surgery [9, 10].

The aim of this prospective, randomized, blinded clinical trial was to assess effect of propofol versus isoflurane anesthesia on alveolar and systemic immune modulation during thoracic surgery. We hypothesized that both propofol and isoflurane have similar effects on lung and systemic immunity during thoracic surgery.

## 2. Material and Methods

The current study is a prospective, randomized, double-blinded study done on 50 adult patients ASA I-III undergoing elective open thoracic surgery using one-lung ventilation. A written informed consent was obtained from the patients, and institutional review board approval was obtained. All patients were thoroughly evaluated and investigated to ensure fitness for surgery. ECG, echocardiography, arterial blood gases, and chest CAT scanning were performed for all patients. Exclusion criteria included significant lung diseases {forced expiratory volume in 1 s (FEV1) or vital capacity (VC) < 50% of the predicted values}, heart failure or mean pulmonary artery pressure (MPAP) > 30 mmHg, coagulation disorders or a history of preoperative immuno-suppressant medications. All the operations were performed by the same surgical team. Epidural analgesia was done before induction of anesthesia at the T4–T7 level by inserting an epidural catheter (Braun perifix 18 ba and a microporous filter). 

A test dose of 4 mL 1% lidocaine with epinephrine 5 *μ*g/mL was used for verifying intrathecal or intravascular injection, respectively. Epidural block activation was performed by injecting 12 mL of bupivacaine hydrochloride 0.25%. In addition, 4 mL was injected 2 hours later as a maintenance dose and every hour thereafter for postoperative epidural analgesia. The patients were randomly allocated to receive propofol (*n* = 25) or isoflurane (*n* = 25) anesthesia using a random number table generated by Microsoft Excel. An independent statistician was assigned to perform central randomization to ensure proper concealment of the study management from the patients and investigators until the release of the final statistical results. The patients were given 1-2 mg of midazolam i.v. as a premedication about 10–20 min. before induction of anesthesia. 

In the propofol group, general anesthesia was induced with propofol 1.5–2 mg kg^−1^ and fentanyl 3 *μ*g kg^−1^ in addition to cis-atracurium 0.1 mg kg^−1^ for tracheal intubation. Maintenance of anesthesia was done with a continuous infusion of propofol 4–6 mg kg^−1^ h^−1^ and cis-atracurium 2 *μ*g kg^−1^ min^−1^. In the isoflurane group, anesthesia was induced as before but maintained with isoflurane 1MAC, and cis-atracurium 2 *μ*g kg^−1^ min^−1^. In the operating room, a radial arterial catheter and multiple peripheral intravenous catheters were inserted. The patients were monitored by continuous ECG, pulse oxymetry, capnography, central venous pressure, and invasive blood pressure during the whole procedure. Furthermore, arterial blood-gas tensions were measured every hour during surgery and every four hours thereafter. A left-sided double-lumen endobronchial tube (DLT, Broncho-Cathw 39 or 41 Ch., Mallinckrodt Medical Ltd, Ireland) was inserted, and a fiberoptic bronchoscope was used to confirm proper position of the tube. The patients were ventilated with a VT of 10 mL kg^−1^, fraction of inspired oxygen (FIO_2_) of 0.60 in air; the respiratory rate was modulated to keep the end-tidal CO_2_ at normal values of 35–45 mmHg, and a PEEP of 5 cm H_2_O was applied. During one-lung ventilation (OLV), A VT of 10 mL kg^−1^ and FIO_2_ of 0.8 to 1.0 was used to achieve a PaO_2_ of 80 mmHg, and the respiratory rate was adjusted to keep the end-tidal CO_2_ at 35–45 mmHg. The peak inspiratory pressure limit was set at 30 cm H_2_O. Fluid and blood replacements were adjusted to maintain patient hematocrit value above 30%. Fluid warming and forced air warming (Bair Hugger) were used to maintain normothermia. One analyst was blinded in respect to the drug under study during the procedure by covering the lines, infusion pump, gas analyzer, vaporizer, and by numeric codes during the whole process of data evaluation. In addition, physicians who were responsible for postoperative care of these patients and for their discharges from ICU and hospital were effectively blinded to the study protocol. The patients were kept in the ICU till restoration to their preoperative physiological homeostasis including stable hemodynamics, adequate ventilation, normothermia and satisfactory pain control. Discharging from hospital was guided by the ability to ambulate independently and to tolerate oral feeding.

Bronchoalveolar lavage (BAL) was done by passing the fiberoptic bronchoscope through the endobronchial tube. The tip of the scope was wedged into a segmental bronchus of either the right middle lobe or left lower lobe of the ventilated lung. Physiological saline 0.9% was used for BAL in 20 mL portions that were sequentially injected and suctioned, and the total volume instilled was 100 mL of which 55–60% was recovered and both groups were comparable regarding the return volume.

The primary outcome measures were alveolar and plasma concentrations of interleukin-8(IL-8) and tumour necrosis factor-*α* (TNF-*α*). Secondary outcome measures were alveolar and plasma concentrations of both malondialdehyde (MDA) and superoxide dismutase (SOD) and changes in alveolar albumin concentrations and cell numbers. These parameters were recorded at three time points, after induction of anesthesia (*T*_0_), 1h after OLV (*T*_1_), 1 h after surgery (*T*_2_). Alveolar and blood samples were immediately centrifuged and the supernatants separated and placed in Eppendorf tubes and frozen at −80°C until assay. Commercial kits were used for the determination of IL-8 and TNF-*α* (Test-Pig ELISA Kit; Biomed, Diepenbeek, Belgium) based on enzyme-linked immunosorbent assay (ELISA). Recordings were done on a plate reader (GEST, General Elisa System Technology, Menarini Labs, Badalona, Spain) for the automatic ELISA technique in triplicate. Serum MDA assay: the measurement obtained may reflect some combination of free and bound MDA already present in the sample [11]. Measurement of SOD activity was performed through detection of pyrogallol auto-oxidation [12]. Albumin concentration in the alveoli was determined by using nephelometry, whereas changes in alveolar cell numbers were evaluated electronically by using a Coulter Counter (Industrial model D). 

Furthermore, arterial blood gases and airway pressures were recorded at the following time points: during two-lung ventilation, during one-lung ventilation, and after one-lung ventilation. In addition, postoperative outcome was reported in both groups regarding incidence of atelectasis requiring bronchoscopy, pneumonia, ARDS (adult respiratory distress syndrome), respiratory failure requiring ventilation, oxygen need at hospital discharge, 30-day mortality, total number of complications, in addition to ICU and hospital stay times.

## 3. Statistics

With a 2-sided type I error of 5% and study power at 80%, a mean sample size of 25 patients in each group was found enough to detect differences in alveolar and plasma cytokine concentrations between propofol and isoflurane anesthesia according to a previous study [10]. Continuous variables were reported as mean (standard deviation) and categorical variables were expressed as percentages. Statistical analyses were performed using Statistica for Windows version 10.0 software. The Kolmogorov-Smirnov test was used to verify normal distribution of data. Distribution of residuals testing was performed to confirm that ANOVA was appropriate to our data. Data were analyzed on an intention to treat basis using two-way analysis of variance (ANOVA) for repeated measures. This was followed by Student-Newman-Keuls test, if a difference between groups had been detected. Changes over time in nonnormally distributed data sets were analyzed by Friedman repeated measures ANOVA on ranks. *P* < 0.05 was considered statistically significant.

## 4. Results

Both groups were comparable regarding the patients^,^ demographic and operative data (Table 1). 

Alveolar and plasma concentrations of IL-8, TNF-*α* increased significantly in both groups, however, they were significantly lower in the isoflurane group (Tables 2 and 3). Moreover, alveolar albumin concentrations and cell numbers increased significantly in both groups but were significantly lower in the isoflurane group (Table 4). 

Both alveolar and plasma MDA levels increased significantly in patients exposed to isoflurane and were significantly higher than in patients receiving propofol who developed decrease in MDA levels. Furthermore, alveolar and plasma SOD levels increased significantly in the propofol group whereas they showed no significant change in patients exposed to isoflurane (Tables 2 and 3).

Both groups were comparable in respect to arterial blood gases and airway pressures during surgery (Table 5).

Total number of complications was significantly lower in the isoflurane group and both ICU and hospital stay time were significantly lower in the isoflurane group (Table 6).

## 5. Discussion

The current study has demonstrated that OLV during thoracic surgery induced alveolar and systemic inflammatory reaction. This immune reaction was less in patients exposed to isoflurane anesthesia when compared to propofol anesthesia as indicated by lower alveolar and plasma concentrations of IL-8 and TNF-*α*. Furthermore, alveolar albumin concentrations and cell numbers were less in patients who received isoflurane anesthesia. However, MDA was higher and SOD was less in patients exposed to isoflurane. Anesthesia, surgery, and OLV induce an inflammatory response in alveolar macrophages that lead to release of proinflammatory cytokines into alveoli and systemic circulation [13]. These cytokines have various actions that include immune modulations and control of tissue infection, injury, and healing [14]. Controlled release of IL-8, TNF-*α* play an important role in fighting against infection; however, the uncontrolled release of these cytokines triggers massive influx of neutrophils and granulocytes with subsequent lung injury and dysfunction [15]. MDA is an indicator of lipid peroxidation, whereas SOD is an antioxidant enzyme that helps in scavenging free radicals which play a role in tissue injury [16]. 

The protective effects of inhalational anesthetics on the heart have been approved, however, there are no enough data regarding the potential benefit to the lung during thoracic surgery [17]. Our findings were matching with the results stated by Cho and colleagues [9], who reported that volatile anesthetics attenuate alveolar and systemic inflammatory response triggered by the heart lung machine. In another study [18], alveolar secretion of inflammatory cytokines in primary culture has been attenuated by inhalational anesthetics. Similarly, De Conno and coworkers [19], reported anti-inflammatory effect of the volatile anesthetic sevoflurane at the lung in patients undergoing thoracic surgery and linked this effect to improved postoperative outcome and 50% reduction of postoperative complications related to lung injury when compared with propofol. In another study [20], volatile anesthetic attenuated the release of inflammatory cytokines from the alveoli in a model of lipopolysaccharide-induced injury in rats. Furthermore, Reuterhaus and coworkers [21] demonstrated ability of isoflurane in reducing release of inflammatory mediators in an endotoxin-induced lung injury model. 

In contrast to our results, other studies have demonstrated augmentation of the release of alveolar inflammatory mediators by volatile anesthetics during mechanical ventilation in both rats and pigs [22, 23]. These contradictory findings may be explained by variations in patient populations, use of different inhalational anesthetics and variations in the exposure time, different methods of biochemical analysis, and absence of settled dilution markers. Furthermore, the interpretation of alveolar inflammatory mediators in the BAL fluid is debatable and difficult as an unknown fraction of the mediators remains inside the cells in addition to the possibility of cell injury during the procedure of BAL. 

In our study, MDA levels were significantly higher in patients exposed to isoflurane whereas SOD levels were significantly higher in the propofol group which imply increased antioxidant capacity in patients receiving propofol. However, a high antioxidant capacity may not be a nice event if it denotes a reaction to an increased oxidative stress. Therefore, evaluation of the antioxidant status must be linked in a dynamic manner to the circumstances under which it is measured. It should be noticed that increased inflammatory mediators in alveoli may not bear major clinical implications in healthy subjects; however in patients with expected high levels of inflammatory cytokines like cancer and chronic renal failure patients, the proper selection of the anesthetic may be highly important.

In conclusion, our study has shown anti-inflammatory effect of the volatile anesthetic isoflurane in patients undergoing thoracic surgery with OLV that may have clinical implications in situations of expected high inflammatory mediators like cancer patients. Furthermore, the overall postoperative outcome was significantly better in patients receiving isoflurane anesthesia.

## Figures and Tables

**Table 1:** Patients' demographic and operative data.

Variable	Group I (Propofol) No. = 25	Group II (Isoflurane) No. = 25
Age (y)	50.3 (13)	48.8 (14)
Sex (M/F)	18/7	17/8
Weight (kg)	77 (7.1)	75 (6.8)
Height (cm)	172 (15)	170 (12)
ASA physical status		
I	4	5
II	19	17
III	2	3
Type of operations:		
Wedge resection	17	16
Lobectomy	6	7
Pneumonectomy	2	2
Operative time (min)	137 (43)	139 (45)
OLV time (min)	80 (34)	78 (36)
Anesthesia time (min)	152 (47)	154 (45)
Transfused units of blood/patients	10/5	11/7

Data are given as mean (SD) or numbers. ASA: American Society of Anesthesiologists; OLV: one-lung ventilation.

**Table 2:** Changes in alveolar IL-8, TNF-*α*, MDA, and SOD in both groups.

Time points	Group I (Propofol) No. = 25				Group II (Isoflurane) No. = 25			
	IL-8 (pg/mL)	TNF-*α* (pg/mL)	MDA (*μ*moL/litre)	SOD (U/g Hb)	IL-8 (pg/mL)	TNF-*α* (pg/mL)	MDA (*μ*moL/litre)	SOD (U/g Hb)
*T*_0_	331 (68)	4.7 (1.9)	0.31 (0.04)	98 (14)	328 (65)	5.0 (2.1)	0.29 (0.06)	96 (12)
*T*_1_	787 (74)^†∗^	36.5 (15.1)^†∗^	0.08 (0.03)^†∗^	119 (16)^†∗^	522 (80)^‡∗^	21.1 (8.1)^‡∗^	0.43 (0.09)^‡∗^	100 (15)*
*T*_2_	954 (78)^†∗^	50.4 (20.4)^†∗^	0.07 (0.02)^†∗^	117 (15)^†∗^	601 (82)^‡∗^	26.3 (12.4^)‡∗^	0.46 (0.13)^‡∗^	101 (14)*

IL-8: interleukin-8; TNF-*α*: tumour necrosis factor-*α*; MDA: malondialdehyde; SOD: superoxide dismutase. Data are expressed as mean (SD). ^†^ *P* < 0.05 within the propofol group, ^‡^ *P* < 0.05 within the isoflurane group, *P < 0.05 between both groups.

**Table 3:** Changes in plasma IL-8, TNF-*α*, MDA, and SOD in both groups.

Time points	Group I (Propofol) No. = 25				Group II (Isoflurane) No. = 25			
	IL-8 (pg/mL)	TNF-*α* (pg/mL)	MDA (*μ*moL/litre)	SOD (U/g Hb)	IL-8 (pg/mL)	TNF-*α* (pg/mL)	MDA (*μ*moL/litre)	SOD (U/g Hb)
*T*_0_	2.5 (0.4)	21 (5)	2.8 (1.2)	532 (54)	2.4 (0.5)	23 (6)	2.7 (1.0)	538 (62)
*T*_1_	16 (2.4)^†∗^	95 (24)^†∗^	1.8 (0.5)^†∗^	644 (58)^†∗^	9.1 (1.4)^‡∗^	77 (19)^‡∗^	4.1 (1.6)^‡∗^	542 (70)*
*T*_2_	13.4 (2.9)^†∗^	293 (53)^†∗^	1.7 (0.4)^†∗^	640 (57)^†∗^	7.9 (2.4)^‡∗^	151 (41)^‡∗^	4.4 (1.3)^‡∗^	543 (69)*

IL-8: interleukin-8; TNF-*α*: tumour necrosis factor-*α*; MDA: malondialdehyde; SOD: superoxide dismutase. Data are expressed as mean (SD). ^†^ *P* < 0.05 within the propofol group, ^‡^ *P* < 0.05 within the isoflurane group, *P < 0.05 between both groups.

**Table 4:** Changes in alveolar albumin concentrations and cell numbers in both groups.

Time points	Group I (Propofol) No. = 25		Group II (Isoflurane) No. = 25	
	albumin (*μ*g/mL)	Cells (×10^6^/mL)	albumin (*μ*g/mL)	Cells (×10^6^/mL)
*T*_0_	20 (9)	0.07 (0.05)	19 (8)	0.06 (0.04)
*T*_1_	101 (31)^†∗^	0.19 (0.09)^†∗^	51 (15)^‡∗^	0.11 (0.07)^‡∗^
*T*_2_	121 (44)^†∗^	0.21 (0.10)^†∗^	78 (30)^‡∗^	0.12 (0.06)^‡∗^

Data are expressed as mean (SD). ^†^ *P* < 0.05 within the propofol group, ^‡^ *P* < 0.05 within the isoflurane group, *P < 0.05 between both groups.

**Table 5:** Arterial blood gases and airway pressures in both groups.

Variable	Group I (Propofol) No. = 25			Group II (Isoflurane) No. = 25		
	During TLV	During OLV	After OLV	During TLV	During OLV	After OLV
PaO_2_ (mmHg)	268 (88)	115 (54)	284 (87)	265 (86)	111 (51)	278 (88)
PaCO_2_ (mmHg)	35.6 (3.9)	36.2 (4.2)	35.5 (4.0)	36.4 (3.8)	36.9 (3.9)	36.1 (4.0)
pH	7.41 (0.04)	7.40 (0.04)	7.41 (0.04)	7.40 (0.04)	7.40 (0.05)	7.40 (0.04)
SaO_2_ (%)	99.1 (1.3)	96.2 (2.5)	99.3 (1.2)	99.0 (1.1)	95.7 (2.2)	98.7 (1.5)
Ppeak (cm H_2_O)	17.6 (5.1)	24.4 (5.3)	17.2 (3.9)	18.2 (5.4)	25.2 (4,9)	17.6 (4.1)
Pmean (cm H_2_O)	6.1 (2.0)	8.9 (2.2)	6.0 (1.2)	6.4 (1.8)	9.2 (2.1)	6.3 (1.3)
Pplateau (cm H_2_O)	13.8 (2.9)	17.1 (3.9)	13.6 (2.2)	14.0 (2.7)	17.5 (3.8)	13.9 (2.4)

TLV: two-lung ventilation; OLV: one-lung ventilation; PaO_2_: arterial oxygen tension; PaCO_2_: arterial carbon dioxide tension; SaO_2_: arterial oxygen saturation; Ppeak: peak inspiratory pressure; Pmean: mean inspiratory pressure; Pplateau: plateau inspiratory pressure. Data are expressed as mean (SD). *P* > 0.05 denotes statistical insignificance.

**Table 6:** Postoperative outcome in both groups.

Variable	Group I (Propofol) No. = 25	Group II (Isoflurane) No. = 25	*P* value
Atelectasis requiring bronchoscopy	5	2	0.22
Pneumonia	4	1	0.16
ARDS	0	0	N/A
Respiratory failure requiring ventilation	1	0	0.31
Oxygen need at hospital discharge	0	0	N/A
30-day mortality	0	0	N/A
Total number of complications	10	3	0.02*
ICU stay (hours)	37 (7)	26 (8)	0.02*
Hospital stay (days)	11 (5)	7 (4)	0.03*

ARDS: Adult respiratory distress syndrome; ICU: Intensive care unit; N/A: Not applicable.

Data are expressed as numbers or as mean (SD). *P < 0.05 between both groups.

## References

[1] Schilling T, Kozian A, Huth C (2005). The pulmonary immune effects of mechanical ventilation in patients undergoing thoracic surgery. *Anesthesia and Analgesia*.

[2] Frass OM, Bühling F, Täger M (2001). Antioxidant and antiprotease status in peripheral blood and BAL fluid after cardiopulmonary bypass. *Chest*.

[3] Kotani N, Lin CY, Wang JS (1995). Loss of alveolar macrophages during anesthesia and operation in humans. *Anesthesia and Analgesia*.

[4] Dreyfuss D, Saumon G (1998). Ventilator-induced lung injury: lessons from experimental studies. *American Journal of Respiratory and Critical Care Medicine*.

[5] Kotani N, Hashimoto H, Sessler DI (2000). Smoking decreases alveolar macrophage function during anesthesia and surgery. *Anesthesiology*.

[6] Craig SR, Leaver HA, Yap PL, Pugh GC, Walker WS (2001). Acute phase responses following minimal access and conventional thoracic surgery. *European Journal of Cardio-Thoracic Surgery*.

[7] Takao Y, Mikawa K, Nishina K, Obara H (2005). Attenuation of acute lung injury with propofol in endotoxemia. *Anesthesia and Analgesia*.

[8] Kotani N, Takahashi S, Sessler DI (1999). Volatile anesthetics augment expression of proinflammatory cytokines in rat alveolar macrophages during mechanical ventilation. *Anesthesiology*.

[9] Cho EJ, Yoon JH, Hong SJ, Lee SH, Sim SB (2009). The effects of sevoflurane on systemic and pulmonary inflammatory responses after cardiopulmonary bypass. *Journal of Cardiothoracic and Vascular Anesthesia*.

[10] Schilling T, Kozian A, Kretzschmar M (2007). Effects of propofol and desflurane anaesthesia on the alveolar inflammatory response to one-lung ventilation. *British Journal of Anaesthesia*.

[11] Wong SHY, Knight JA, Hopfer SM, Zaharia O, Leach CN, Sunderman FW (1987). Lipoperoxides in plasma as measured by liquid-chromatographic separation of malondialdehyde-thiobarbituric acid adduct. *Clinical Chemistry*.

[12] Marklund S, Marklund G (1974). Involvement of the superoxide anion radical in the autoxidation of pyrogallol and a convenient assay for superoxide dismutase. *European Journal of Biochemistry*.

[13] Wrigge H, Zinserling J, Stuber F (2000). Effects of mechanical ventilation on release of cytokines into systemic circulation in patients with normal pulmonary function. *Anesthesiology*.

[14] Kotani N, Hashimoto H, Sessler DI (1998). Intraoperative modulation of alveolar macrophage function during isoflurane and propofol anesthesia. *Anesthesiology*.

[15] Kooguchi K, Hashimoto S, Kobayashi A (1998). Role of alveolar macrophages in initiation and regulation of inflammation in Pseudomonas aeruginosa pneumonia. *Infection and Immunity*.

[16] Ahmed MN, Codipilly C, Hogg N, Auten RL (2011). The protective effect of overexpression of extracellular superoxide dismutase on nitric oxide bioavailability in the lung after exposure to hyperoxia stress. *Experimental Lung Research*.

[17] Tanaka K, Ludwig LM, Kersten JR, Pagel PS, Warltier DC (2004). Mechanisms of cardioprotection by volatile anesthetics. *Anesthesiology*.

[18] Giraud O, Molliex S, Rolland C (2003). Halogenated anesthetics reduce interleukin-1 *β*-induced cytokine secretion by rat alveolar type II cells in primary culture. *Anesthesiology*.

[19] De Conno E, Steurer MP, Wittlinger M (2009). Anesthetic-induced improvement of the inflammatory response to one-lung ventilation. *Anesthesiology*.

[20] Suter D, Spahn DR, Blumenthal S (2007). The immunomodulatory effect of sevoflurane in endotoxin-injured alveolar epithelial cells. *Anesthesia and Analgesia*.

[21] Reutershan J, Chang D, Hayes JK, Ley K (2006). Protective effects of isoflurane pretreatment in endotoxin-induced lung injury. *Anesthesiology*.

[22] Kotani N, Takahashi S, Sessler DI (1999). Volatile anesthetics augment expression of proinflammatory cytokines in rat alveolar macrophages during mechanical ventilation. *Anesthesiology*.

[23] Takala RSK, Soukka HR, Salo MS (2004). Pulmonary inflammatory mediators after sevoflurane and thiopentone anaesthesia in pigs. *Acta Anaesthesiologica Scandinavica*.

